# Supplementary material for: Towards Predicting Gut Microbial Metabolism: Integration of Flux Balance Analysis and Untargeted Metabolomics
Source: Metabolites. 2020 Apr 17;10(4):156. doi: 10.3390/metabo10040156 (PMC7240944; doi:10.3390/metabo10040156)
Supplement: Supplementary file 1 [file metabolites-10-00156-s001.pdf]

metabolites

Supplementary Information

Towards Predicting Gut Microbial Metabolism: Integration of Flux Balance Analysis and Untargeted Metabolomics

Ellen Kuang [1], Matthew Marney [2], Daniel Cuevas [3], Robert A. Edwards [2,3,4] and Erica M. Forsberg [1,2,3,*]

[1] Department of Chemistry and Biochemistry, San Diego State University, San Diego, CA 92182, USA; ekuang@sdsu.edu (E.K.), eforsberg@sdsu.edu (E.M.F.)

[2] Department of Biomedical Informatics, San Diego State University, San Diego, CA 92182, USA; mmarney@gmail.com (M.M.); redwards@sdsu.edu (R.A.E.)

[3] Viral Information Institute, San Diego State University, San Diego, CA 92182, USA; dcuevas08@gmail.com (D.C.)

[4] Department of Biology, San Diego State University, San Diego, CA 92182, USA

* Correspondence: eforsberg@sdsu.edu; Tel.: +1-619-594-5806

2. Results

The output of MS_FBA generates both graphical visualizations (**Figure S1**) and a feature table containing the potential matches between the untargeted metabolomics data and the flux balance analysis metabolite list (**Table S1** and **S2**). These show the molecule name, *m*/*z* value, retention time, peak group (e.g. same retention time window), the difference between the expected and observed M+1 isotope ratio, whether this is considered to be an isotope match (e.g. the isotope must be within 5% of the expected ratio) and the predicted adduct. These are illustrated below for the *C. sedlakii* data in both positive mode reverse phase (**Table S1**) and negative mode HILIC (**Table S2**).

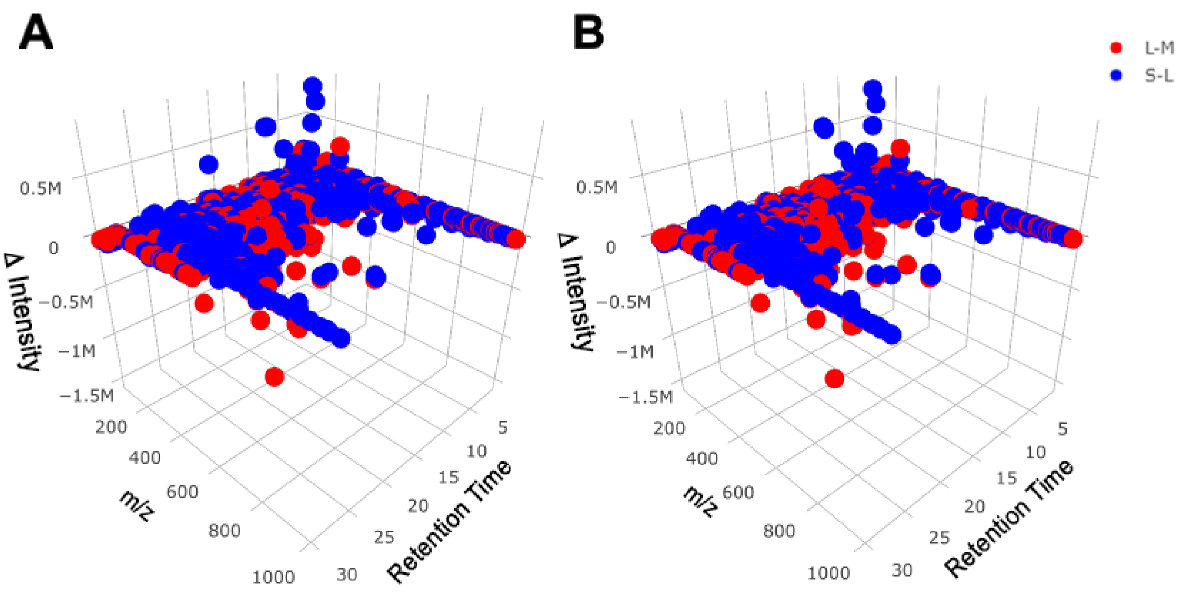

Figure S1. (**a**) This graphical output from MS_FBA shows all 1097 significant features from the XCMS Online results for the reverse phase positive mode run. The red dots show the intensity difference between the late (L) and mid (M) sample points, and the blue shows the intensity difference between stationary (S) to late (L). (**b**) This graph shows all features that were not matched to the compounds from the predicted compound list.

Table S1. Features matched to PyFBA predicted metabolites in the reverse phase run with data acquisition in positive mode.

Compound	m/z	Retention Time (min)	Peak Group	Isotope Difference (%)	Isotope Match	Adduct
D-glutamic acid	148.060	2.027	2	-0.242	TRUE	[M+H]+
L-glutamic acid	148.060	2.027	2	-0.242	TRUE	[M+H]+
O-acetyl-L-serine	148.060	2.027	2	-0.242	TRUE	[M+H]+
D-glyceric acid	148.060	2.027	2	4.885	FALSE	[M+C2H3N+H]+
L-pyroglutamic acid	130.050	2.019	2	-0.291	TRUE	[M+H]+
3-hydroxy-L-1-pyrroline-5-carboxylate	130.050	2.019	2	-0.291	TRUE	[M+H]+
D-glutamic acid	130.050	2.019	2	-0.425	TRUE	[M-H2O+H]+
L-glutamic acid	130.050	2.019	2	-0.425	TRUE	[M-H2O+H]+
O-acetyl-L-serine	130.050	2.019	2	-0.425	TRUE	[M-H2O+H]+
3-oxopropanoic acid	130.050	2.019	2	4.836	FALSE	[M+C2H3N+H]+
Pyruvic acid	130.050	2.019	2	4.836	FALSE	[M+C2H3N+H]+
L-threonine	102.055	2.024	2	-0.419	TRUE	[M-H2O+H]+
L-homoserine	102.055	2.024	2	-0.419	TRUE	[M-H2O+H]+
Acetic acid	102.055	2.024	2	4.842	FALSE	[M+C2H3N+H]+
Glycolaldehyde	102.055	2.024	2	4.842	FALSE	[M+C2H3N+H]+
D-glutamic acid	295.114	2.027	2	-0.336	TRUE	[2M+H]+
L-glutamic acid	295.114	2.027	2	-0.336	TRUE	[2M+H]+
O-acetyl-L-serine	295.114	2.027	2	-0.336	TRUE	[2M+H]+
L-proline	116.071	2.017	2	0.398	TRUE	[M+H]+
Propionic acid	116.071	2.017	2	5.525	FALSE	[M+C2H3N+H]+
(S)-lactaldehyde	116.071	2.017	2	5.525	FALSE	[M+C2H3N+H]+
Lactaldehyde	116.071	2.017	2	5.525	FALSE	[M+C2H3N+H]+
L-Arabino-1,4-lactone	131.034	2.023	2	1.228	FALSE	[M-H2O+H]+
Diadenosine tetraphosphate	248.036	2.061	2	-3.550	FALSE	[M+2H]+
Raffinose	527.159	2.002	2	0.710	TRUE	[M+Na]+
Maltotriose	527.159	2.002	2	0.710	TRUE	[M+Na]+

6-O-(alpha-D-galactopyranosyl)-6-O-(alpha-D-galactopyranosyl)-D-glucopyranose	527.159	2.002	2	0.710	TRUE	[M+Na]+
L-aspartic acid	175.071	2.056	2	-2.347	FALSE	[M+C2H3N+H]+
Urocanic acid	175.071	2.056	2	-7.218	FALSE	[M+2H2O+H]+
D-glyceric acid	170.042	2.000	2	-0.898	TRUE	[M+C2H3N+Na]+
D-glutamic acid	170.042	2.000	2	-6.025	FALSE	[M+Na]+
L-glutamic acid	170.042	2.000	2	-6.025	FALSE	[M+Na]+
O-acetyl-L-serine	170.042	2.000	2	-6.025	FALSE	[M+Na]+
Maltotetraose	689.212	2.045	2	-27.201	FALSE	[M+Na]+
Stachyose	689.212	2.045	2	-27.201	FALSE	[M+Na]+
Melibiose	343.124	2.048	2	-13.620	FALSE	[M+H]+
Sucrose	343.124	2.048	2	-13.620	FALSE	[M+H]+
Alpha-lactose	343.124	2.048	2	-13.620	FALSE	[M+H]+
Trehalose	343.124	2.048	2	-13.620	FALSE	[M+H]+
5,6-Dimethylbenzimidazole	179.049	3.367	5	-1.853	FALSE	[M+C2H3N+H]+
Salicin	309.094	3.366	5	-14.513	FALSE	[M+Na]+
dUMP	309.050	3.391	5	-10.900	FALSE	[M+H]+
Gamma-glutamylcysteine	233.059	3.366	5	-0.798	TRUE	[M-H2O+H]+
Aspartyl phosphate	255.039	3.403	5	-2.472	FALSE	[M+C2H3N+H]+
3-oxo-O-phosphono-L-homoserine	255.039	3.403	5	-2.472	FALSE	[M+C2H3N+H]+
1-Stearoyl-sn-glycero-3-phosphate	480.308	20.648	9	5.190	FALSE	[M+C2H3N+H]+
N1-acetylspermidine	188.176	1.569	11	-1.864	FALSE	[M+H]+
N8-acetylspermidine	188.176	1.569	11	-1.864	FALSE	[M+H]+
l-histidinol phosphate	245.054	1.623	11	-7.866	FALSE	[M+H+Na]+
L-beta-lysine	191.077	1.542	11	-7.444	FALSE	[M+2Na-H]+
L-lysine	191.077	1.542	11	-7.444	FALSE	[M+2Na-H]+
Deoxy-TDP	358.039	7.243	12	0.144	TRUE	[M+C2H3N+Na]+
5'-phosphoribosylformyl-	358.039	7.243	12	-10.224	FALSE	[M+2Na-H]+

glycinamidine						
1-stearoyl-sn-glycero-3-phosphate	480.308	21.147	15	4.816	FALSE	[M+C2H3N+H]+
1-stearoyl-sn-glycero-3-phosphate	502.290	21.147	15	5.318	FALSE	[M+C2H3N+Na]+
N-acetylneuraminic acid	348.070	4.336	16	-0.413	TRUE	[M+K]+
Adenine	136.062	4.331	16	-2.196	FALSE	[M+H]+
dUMP	350.075	4.335	16	-8.337	FALSE	[M+C2H3N+H]+
Pyridoxine phosphate	232.036	4.338	16	-9.436	FALSE	[M-H2O+H]+
SEPHCHC	367.044	4.332	16	-15.646	FALSE	[M+K]+
Cytidine-5'-diphosphate	404.028	4.327	16	-11.406	FALSE	[M+H]+
Adenine	136.062	2.230	19	-1.270	TRUE	[M+H]+
Betaine	118.086	2.219	19	0.033	TRUE	[M+H]+
L-valine	118.086	2.219	19	0.033	TRUE	[M+H]+
1,2-propanediol	118.086	2.219	19	5.160	FALSE	[M+C2H3N+H]+
L-aspartic acid	134.045	2.229	19	-4.910	FALSE	[M+H]+
Palmitic acid	257.248	2.197	19	-0.434	TRUE	[M+H]+
Glyoxylic acid	116.034	2.207	19	0.278	TRUE	[M+C2H3N+H]+
L-aspartic acid	116.034	2.207	19	-4.983	FALSE	[M-H2O+H]+
Maltohexaose	991.335	2.235	19	-4.719	FALSE	[M+H]+
Butyric acid	130.087	1.944	20	-1.913	FALSE	[M+C2H3N+H]+
Pyridoxamine	210.124	1.946	20	-7.017	FALSE	[M+C2H3N+H]+
Cytidine	244.093	1.942	20	-11.155	FALSE	[M+H]+
Gamma-glutamyl-beta-cyano-alanine	244.093	1.942	20	-11.155	FALSE	[M+H]+
Melibiose	365.106	1.943	20	0.102	TRUE	[M+Na]+
Sucrose	365.106	1.943	20	0.102	TRUE	[M+Na]+
Alpha-lactose	365.106	1.943	20	0.102	TRUE	[M+Na]+
Trehalose	365.106	1.943	20	0.102	TRUE	[M+Na]+
Methanesulfonic acid	192.983	1.940	20	-4.070	FALSE	[2M+H]+
Guanine	152.057	2.432	21	-1.686	FALSE	[M+H]+

Butyric acid	112.051	2.441	21	0.100	TRUE	[M+H+Na]+
Cytosine	112.051	2.441	21	-0.934	TRUE	[M+H]+
5'-Cytidylic acid	324.059	2.438	21	0.181	TRUE	[M+H]+
Cytidine 3'-monophosphate	324.059	2.438	21	0.181	TRUE	[M+H]+
L-pyroglutamic acid	166.072	2.455	21	0.823	TRUE	[M+2H2O+H]+
3-hydroxy-L-1-pyrroline-5-carboxylate	166.072	2.455	21	0.823	TRUE	[M+2H2O+H]+
Nicotinamide	123.055	3.874	27	-0.675	TRUE	[M+H]+
Nicotinamide dinucleotide	664.116	3.867	27	1.863	FALSE	[M+H]+
ADP-D-ribose	542.068	3.867	27	-0.945	TRUE	[M-H2O+H]+
Phosphoribosyl-AMP	542.068	3.867	27	-0.945	TRUE	[M-H2O+H]+
N-succinyl-L-glutamate 5-semialdehyde	232.083	3.874	27	2.199	FALSE	[M+H]+
UDP-glucuronic acid	622.066	3.868	27	-15.300	FALSE	[M+C2H3N+H]+
2'-deoxyuridine	252.073	3.936	27	-10.775	FALSE	[M+H+Na]+
3-oxopropanoic acid	130.050	4.697	30	-0.825	TRUE	[M+C2H3N+H]+
Pyruvic acid	130.050	4.697	30	-0.825	TRUE	[M+C2H3N+H]+
L-pyroglutamic acid	130.050	4.697	30	-5.952	FALSE	[M+H]+
3-hydroxy-L-1-pyrroline-5-carboxylate	130.050	4.697	30	-5.952	FALSE	[M+H]+
D-glutamic acid	130.050	4.697	30	-6.086	FALSE	[M-H2O+H]+
L-glutamic acid	130.050	4.697	30	-6.086	FALSE	[M-H2O+H]+
O-acetyl-L-serine	130.050	4.697	30	-6.086	FALSE	[M-H2O+H]+
Sulfoacetic acid	177.007	2.106	34	-3.052	FALSE	[M+2H2O+H]+
Urocanic acid	177.007	2.106	34	-7.363	FALSE	[M+K]+
Maltotetraose	667.229	2.122	34	0.558	TRUE	[M+H]+
Stachyose	667.229	2.122	34	0.558	TRUE	[M+H]+
Glycerol	115.037	2.101	34	-3.446	FALSE	[M+Na]+
Thiamine pyrophosphate	425.045	2.139	34	-15.698	FALSE	[M+H]+
L-allysine	187.108	2.150	34	-4.515	FALSE	[M+C2H3N+H]+
6-amino-2-oxohexanoic acid	187.108	2.150	34	-4.515	FALSE	[M+C2H3N+H]+

L-Arabino-1,4-lactone	297.083	23.204	37	15.056	FALSE	[2M+H]+
Folic acid	464.131	23.201	37	-23.550	FALSE	[M+Na]+
Cifostodine	306.049	3.282	38	-0.498	TRUE	[M+H]+
5'-Cytidylic acid	306.049	3.282	38	-0.631	TRUE	[M-H2O+H]+
Cytidine 3'-monophosphate	306.049	3.282	38	-0.631	TRUE	[M-H2O+H]+
Butyric acid	112.051	3.289	38	0.100	TRUE	[M+H+Na]+
Cytosine	112.051	3.289	38	-0.934	TRUE	[M+H]+
Butyraldehyde	145.122	16.690	40	-8.889	FALSE	[2M+H]+
Uroporphyrinogen III	427.265	17.918	42	10.303	FALSE	[M+Na]+
Uroporphyrinogen I	427.265	17.918	42	10.303	FALSE	[M+Na]+
2',3'-cyclic UMP	329.016	17.886	42	-10.889	FALSE	[M+Na]+
2',3'-cyclic UMP	307.034	17.672	43	-0.367	TRUE	[M+H]+
Pseudouridine 5'-phosphate	307.034	17.672	43	-0.501	TRUE	[M-H2O+H]+
3'-uridylic acid	307.034	17.672	43	-0.501	TRUE	[M-H2O+H]+
Uridine 5'-monophosphate	307.034	17.672	43	-0.501	TRUE	[M-H2O+H]+
Anthranilic acid	120.044	10.241	45	-8.147	FALSE	[M-H2O+H]+
4-aminobenzoic acid	120.044	10.241	45	-8.147	FALSE	[M-H2O+H]+
L-arabino-1,4-lactone	149.045	10.247	45	-5.671	FALSE	[M+H]+
sn-Glycerol 3-phosphate	173.021	10.163	45	-3.560	FALSE	[M+H]+
Guanine	152.057	10.227	45	-7.329	FALSE	[M+H]+
Acetohydroxybutyrate	179.021	3.501	46	-4.069	FALSE	[M+C2H3N+H]+
2-dehydropantoate	179.021	3.501	46	-4.069	FALSE	[M+C2H3N+H]+
(R)-3-hydroxy-3-methyl-2-oxopentanoic acid	179.021	3.501	46	-4.069	FALSE	[M+C2H3N+H]+
L-tyrosine	182.081	2.666	48	0.013	TRUE	[M+H]+
Glutathione	182.081	2.666	48	6.859	FALSE	[M-H2O+H]+
3-Hydroxycinnamic acid	165.055	2.667	48	0.871	TRUE	[M+H]+
Phenylpyruvic acid	165.055	2.667	48	0.871	TRUE	[M+H]+
Carbamoylphosphate	163.973	2.683	48	-1.685	FALSE	[M+Na]+
S-succinyldihydrolipoamide	346.055	16.242	49	-3.703	FALSE	[M+K]+

Guanine	152.057	16.242	49	-1.714	FALSE	[M+H]+
Altronic acid	219.048	16.165	49	-6.884	FALSE	[M+Na]+
D-galactonic acid	219.048	16.165	49	-6.884	FALSE	[M+Na]+
Gluconic acid	219.048	16.165	49	-6.884	FALSE	[M+Na]+
D-Mannonic acid	219.048	16.165	49	-6.884	FALSE	[M+Na]+
Anthranilic acid	138.055	7.193	55	-8.074	FALSE	[M+H]+
4-aminobenzoic acid	138.055	7.193	55	-8.074	FALSE	[M+H]+
Pyridoxal	168.066	7.196	55	-9.215	FALSE	[M+H]+
L-phenylalanine	166.086	4.046	56	-0.005	TRUE	[M+H]+
Salicyl alcohol	166.086	4.046	56	5.122	FALSE	[M+C2H3N+H]+
4-hydroxybenzyl alcohol	166.086	4.046	56	5.122	FALSE	[M+C2H3N+H]+
Melibiose	325.113	2.359	59	-0.036	TRUE	[M-H2O+H]+
Sucrose	325.113	2.359	59	-0.036	TRUE	[M-H2O+H]+
Alpha-lactose	325.113	2.359	59	-0.036	TRUE	[M-H2O+H]+
Trehalose	325.113	2.359	59	-0.036	TRUE	[M-H2O+H]+
Raffinose	487.166	2.345	59	-0.968	TRUE	[M-H2O+H]+
Maltotriose	487.166	2.345	59	-0.968	TRUE	[M-H2O+H]+
Manninotriose	487.166	2.345	59	-0.968	TRUE	[M-H2O+H]+
Urocanic acid	139.050	2.277	59	-1.313	TRUE	[M+H]+
Imidazol-4-one-5-propionic acid	139.050	2.277	59	-1.447	TRUE	[M-H2O+H]+
S-adenosylmethioninamine	258.145	2.289	59	5.077	FALSE	[M+Na]+
Maltotetraose	649.219	2.343	59	-0.784	TRUE	[M-H2O+H]+
Stachyose	649.219	2.343	59	-0.784	TRUE	[M-H2O+H]+
D-galactose	163.060	2.333	59	0.029	TRUE	[M-H2O+H]+
Scyllo-inositol	163.060	2.333	59	0.029	TRUE	[M-H2O+H]+
Beta-D-glucose	163.060	2.333	59	0.029	TRUE	[M-H2O+H]+
D-Glucose	163.060	2.333	59	0.029	TRUE	[M-H2O+H]+
Aldehydo-D-galactose	163.060	2.333	59	0.029	TRUE	[M-H2O+H]+
D-Mannose	163.060	2.333	59	0.029	TRUE	[M-H2O+H]+

D-fructofuranose	163.060	2.333	59	0.029	TRUE	[M-H2O+H]+
Demethylmenaquinone	163.060	2.333	59	3.943	FALSE	[M+C2H3N+Na]+
Methylglyoxal	145.050	2.311	59	-6.713	FALSE	[2M+H]+
Delta1-piperideine-2-carboxylate	169.097	2.283	59	-4.454	FALSE	[M+C2H3N+H]+
(S)-2,3,4,5-tetrahydropyridine-2-carboxylic acid	169.097	2.283	59	-4.454	FALSE	[M+C2H3N+H]+
Pyridoxamine	169.097	2.283	59	-9.581	FALSE	[M+H]+
L-leucine	132.102	2.718	61	0.132	TRUE	[M+H]+
L-isoleucine	132.102	2.718	61	0.132	TRUE	[M+H]+
D-serine	147.077	1.887	67	4.354	FALSE	[M+C2H3N+H]+
L-serine	147.077	1.887	67	4.354	FALSE	[M+C2H3N+H]+
Glucosamine	180.087	1.918	67	0.907	TRUE	[M+H]+
(2R,6S)-2,6-diamino-heptanedioic acid	191.103	1.881	67	-8.600	FALSE	[M+H]+
L-threonine	120.066	1.878	67	-4.895	FALSE	[M+H]+
L-homoserine	120.066	1.878	67	-4.895	FALSE	[M+H]+
D-serine	169.058	1.842	67	-1.240	TRUE	[M+C2H3N+Na]+
L-serine	169.058	1.842	67	-1.240	TRUE	[M+C2H3N+Na]+
Thymine	171.014	1.908	67	-6.272	FALSE	[M+2Na-H]+
(S)-2-amino-4-oxobutanoic acid	159.077	1.903	67	-2.309	FALSE	[M+C2H3N+H]+
L-2-amino-3-oxobutanoic acid	159.077	1.903	67	-2.309	FALSE	[M+C2H3N+H]+
Nicotinamide	159.077	1.903	67	-7.180	FALSE	[M+2H2O+H]+
2-aminoacetaldehyde	101.071	1.872	67	-0.049	TRUE	[M+C2H3N+H]+
Delta1-piperideine-2-carboxylate	128.071	1.883	67	-7.017	FALSE	[M+H]+
(S)-2,3,4,5-tetrahydropyridine-2-carboxylic acid	128.071	1.883	67	-7.017	FALSE	[M+H]+
L-allysine	128.071	1.883	67	-7.151	FALSE	[M-H2O+H]+
6-amino-2-oxohexanoic acid	128.071	1.883	67	-7.151	FALSE	[M-H2O+H]+
L-Glutamate 5-semialdehyde	173.092	1.884	67	-3.412	FALSE	[M+C2H3N+H]+

L-Hydroxyproline	173.092	1.884	67	-3.412	FALSE	[M+C2H3N+H]+
Glutamate-1-semialdehyde	173.092	1.884	67	-3.412	FALSE	[M+C2H3N+H]+
5-aminolevulinic acid	173.092	1.884	67	-3.412	FALSE	[M+C2H3N+H]+
(2R,6S)-2,6-diamino-heptanedioic acid	173.092	1.884	67	-8.672	FALSE	[M-H2O+H]+
Maltitol	173.092	1.884	67	-13.631	FALSE	[M+2H]+
Histidinal	176.103	1.903	67	-7.584	FALSE	[M+2H2O+H]+
Toxopyrimidine	176.103	1.903	67	-7.584	FALSE	[M+2H2O+H]+
L-citrulline	176.103	1.903	67	-7.839	FALSE	[M+H]+
Alpha-D-glucosamine 1-phosphate	260.053	1.870	67	-7.303	FALSE	[M+H]+
D-glucosamine 6-phosphate	260.053	1.870	67	-7.303	FALSE	[M+H]+
N-acetyl-D-Glucosamine	260.053	1.870	67	-9.410	FALSE	[M+K]+
N-acetyl-D-Mannosamine	260.053	1.870	67	-9.410	FALSE	[M+K]+
5'-phosphoribosylformylglycinamidine	314.077	1.884	67	-10.224	FALSE	[M+H]+
O-acetyl-L-homoserine	162.076	1.873	67	-7.116	FALSE	[M+H]+
Glucosamine	162.076	1.873	67	-7.250	FALSE	[M-H2O+H]+
L-alanyl-L-leucine	239.162	18.147	71	2.994	FALSE	[M+2H2O+H]+
L-phenylalanine	166.086	7.943	74	1.843	FALSE	[M+H]+
Salicyl alcohol	166.086	7.943	74	6.970	FALSE	[M+C2H3N+H]+
4-hydroxybenzyl alcohol	166.086	7.943	74	6.970	FALSE	[M+C2H3N+H]+
Pyridoxal	168.065	5.874	81	-9.215	FALSE	[M+H]+
Anthranilic acid	138.055	5.874	81	-8.074	FALSE	[M+H]+
4-aminobenzoic acid	138.055	5.874	81	-8.074	FALSE	[M+H]+
L-tryptophan	205.097	6.378	83	0.770	TRUE	[M+H]+
Succinic acid	141.016	3.777	84	3.009	FALSE	[M+Na]+
Succinic acid	119.034	3.781	84	-4.530	FALSE	[M+H]+
L-galactitol-1-phosphate	263.053	8.889	92	-0.079	TRUE	[M+H]+
Mannitol-1-phosphate	263.053	8.889	92	-0.079	TRUE	[M+H]+
Sorbitol-6-phosphate	263.053	8.889	92	-0.079	TRUE	[M+H]+

Iminoaspartate	263.053	8.889	92	-2.881	FALSE	[2M+H]+
2-(3-Carboxypropionyl)-6-hydroxy-cyclohexa-2,4-diene carboxylic acid	263.053	8.889	92	-5.342	FALSE	[M+Na]+
L-fuculose	165.076	8.934	92	-6.797	FALSE	[M+H]+
L-Rhamnose	165.076	8.934	92	-6.797	FALSE	[M+H]+
L-Fucose	165.076	8.934	92	-6.797	FALSE	[M+H]+
6-deoxy-L-fructose	165.076	8.934	92	-6.797	FALSE	[M+H]+
Dulcitol	165.076	8.934	92	-6.930	FALSE	[M-H2O+H]+
D-Sorbitol	165.076	8.934	92	-6.930	FALSE	[M-H2O+H]+
D-mannitol	165.076	8.934	92	-6.930	FALSE	[M-H2O+H]+
Hydroquinone	147.065	8.932	92	-6.480	FALSE	[M+2H2O+H]+
Pyrocatechol	147.065	8.932	92	-6.480	FALSE	[M+2H2O+H]+
L-fuculose	147.065	8.932	92	-6.869	FALSE	[M-H2O+H]+
L-Rhamnose	147.065	8.932	92	-6.869	FALSE	[M-H2O+H]+
L-Fucose	147.065	8.932	92	-6.869	FALSE	[M-H2O+H]+
6-deoxy-L-fructose	147.065	8.932	92	-6.869	FALSE	[M-H2O+H]+
Beta-D-Glucose 1-phosphate	261.037	8.914	92	-6.960	FALSE	[M+H]+
D-fructose-6-phosphate	261.037	8.914	92	-6.960	FALSE	[M+H]+
D-fructofuranose 1-phosphate	261.037	8.914	92	-6.960	FALSE	[M+H]+
Alpha-D-mannose 1-phosphate	261.037	8.914	92	-6.960	FALSE	[M+H]+
Inositol 3-phosphate	261.037	8.914	92	-6.960	FALSE	[M+H]+
1D-myo-inositol 4-phosphate	261.037	8.914	92	-6.960	FALSE	[M+H]+
Inositol 1-phosphate	261.037	8.914	92	-6.960	FALSE	[M+H]+
D-arabino-3-hexulose 6-phosphate	261.037	8.914	92	-6.960	FALSE	[M+H]+
Galactose-1-phosphate	261.037	8.914	92	-6.960	FALSE	[M+H]+
Mannose-6-phosphate	261.037	8.914	92	-6.960	FALSE	[M+H]+
Glucose-6-phosphate	261.037	8.914	92	-6.960	FALSE	[M+H]+
Glucose-1-phosphate	261.037	8.914	92	-6.960	FALSE	[M+H]+
Beta-D-glucose 6-phosphate	261.037	8.914	92	-6.960	FALSE	[M+H]+

D-tagatose 6-phosphate	261.037	8.914	92	-6.960	FALSE	[M+H]+
D-Galactopyranose 1-phosphate	261.037	8.914	92	-6.960	FALSE	[M+H]+
L-galactitol-1-phosphate	525.098	8.955	92	-13.978	FALSE	[2M+H]+
Mannitol 1-phosphate	525.098	8.955	92	-13.978	FALSE	[2M+H]+
Sorbitol 6-phosphate	525.098	8.955	92	-13.978	FALSE	[2M+H]+
5'-methylthio-D-ribose	225.017	8.947	92	-7.559	FALSE	[M+2Na-H]+
L-arginine	175.119	1.640	93	-8.182	FALSE	[M+H]+
Allophanic acid	128.019	1.364	96	12.387	FALSE	[M+H+Na]+
Butyric acid	130.086	1.475	96	-1.913	FALSE	[M+C2H3N+H]+
L-beta-lysine	147.113	1.453	96	-7.444	FALSE	[M+H]+
L-lysine	147.113	1.453	96	-7.444	FALSE	[M+H]+
1-aminopropan-2-one	147.113	1.453	96	-7.444	FALSE	[2M+H]+
D-pantothenic acid	220.118	5.590	99	6.864	FALSE	[M+H]+
Propionaldehyde	117.091	5.557	99	-6.683	FALSE	[2M+H]+
D-alanyl-D-alanine	161.092	1.786	100	-0.050	TRUE	[M+H]+
L-threonine	161.092	1.786	100	5.077	FALSE	[M+C2H3N+H]+
L-homoserine	161.092	1.786	100	5.077	FALSE	[M+C2H3N+H]+
D-alanine	134.019	1.780	100	-3.754	FALSE	[M+2Na-H]+
L-alanine	134.019	1.780	100	-3.754	FALSE	[M+2Na-H]+
Beta-alanine	134.019	1.780	100	-3.754	FALSE	[M+2Na-H]+
L-threonine	183.074	1.752	100	-2.343	FALSE	[M+C2H3N+Na]+
L-homoserine	183.074	1.752	100	-2.343	FALSE	[M+C2H3N+Na]+
D-alanyl-D-alanine	183.074	1.752	100	-7.470	FALSE	[M+Na]+
Pyridoxamine phosphate	249.064	1.768	100	-9.706	FALSE	[M+H]+
2-phospho-D-glyceric acid	224.958	1.711	100	-3.587	FALSE	[M+K]+
3-phospho-D-glycerate	224.958	1.711	100	-3.587	FALSE	[M+K]+
D-alanine	90.055	1.811	100	-3.754	FALSE	[M+H]+
L-alanine	90.055	1.811	100	-3.754	FALSE	[M+H]+
Beta-alanine	90.055	1.811	100	-3.754	FALSE	[M+H]+

Palmitic acid	257.248	26.859	101	1.564	FALSE	[M+H]+
Palmitic acid	301.212	26.865	101	1.912	FALSE	[M+2Na-H]+
Palmitic acid	239.237	26.852	101	-17.785	FALSE	[M-H2O+H]+
Allophanic acid	168.038	26.856	101	-0.495	TRUE	[M+C2H3N+Na]+
N-acetylneuraminic acid	348.071	6.631	103	0.658	TRUE	[M+K]+
2',3'-cyclic UMP	307.033	18.853	108	-0.334	TRUE	[M+H]+
Pseudouridine 5'-phosphate	307.033	18.853	108	-0.468	TRUE	[M-H2O+H]+
3'-uridylic acid	307.033	18.853	108	-0.468	TRUE	[M-H2O+H]+
Uridine-5'-monophosphate	307.033	18.853	108	-0.468	TRUE	[M-H2O+H]+
2',3'-Cyclic UMP	307.033	18.327	110	-0.644	TRUE	[M+H]+
Pseudouridine 5'-phosphate	307.033	18.327	110	-0.777	TRUE	[M-H2O+H]+
3'-uridylic acid	307.033	18.327	110	-0.777	TRUE	[M-H2O+H]+
Uridine 5'-monophosphate	307.033	18.327	110	-0.777	TRUE	[M-H2O+H]+
Tetradecanoic acid	229.216	24.693	118	0.086	TRUE	[M+H]+
Tetradecanoic acid	211.206	24.687	118	-1.170	TRUE	[M-H2O+H]+
Guanine	152.057	9.649	120	-7.329	FALSE	[M+H]+
Pseudouridine-5'-phosphate	307.033	19.337	124	0.053	TRUE	[M-H2O+H]+
3'-uridylic acid	307.033	19.337	124	0.053	TRUE	[M-H2O+H]+
Uridine 5'-monophosphate	307.033	19.337	124	0.053	TRUE	[M-H2O+H]+
2',3'-cyclic UMP	307.033	19.337	124	0.186	TRUE	[M+H]+
D-Galactose	203.053	1.959	133	0.179	TRUE	[M+Na]+
Scyllo-inositol	203.053	1.959	133	0.179	TRUE	[M+Na]+
Beta-D-glucose	203.053	1.959	133	0.179	TRUE	[M+Na]+
D-Glucose	203.053	1.959	133	0.179	TRUE	[M+Na]+
Aldehydo-D-galactose	203.053	1.959	133	0.179	TRUE	[M+Na]+
D-mannose	203.053	1.959	133	0.179	TRUE	[M+Na]+
D-fructofuranose	203.053	1.959	133	0.179	TRUE	[M+Na]+
Gamma-glutamylcysteine	251.070	3.965	134	-1.614	FALSE	[M+H]+
S-D-lactoylglutathione	380.113	3.962	134	-0.465	TRUE	[M+H]+
Allophanic acid	128.019	3.969	134	-3.047	FALSE	[M+H+Na]+

L-cysteine	122.027	3.964	134	-4.555	FALSE	[M+H]+
Methylthioadenosine	298.097	6.087	135	-1.476	TRUE	[M+H]+
2-hydroxy-3-(phosphonooxy)-propyl myristate	424.246	16.401	136	5.425	FALSE	[M+C2H3N+H]+
(S)-1-pyrroline-5-carboxylate	137.046	13.225	141	-5.914	FALSE	[M+H+Na]+
Hypoxanthine	137.046	13.225	141	-6.949	FALSE	[M+H]+
Phosphoric acid	98.985	11.680	144	-0.175	TRUE	[M+H]+
Acetic acid	98.985	11.680	144	-2.282	FALSE	[M+K]+
Glycolaldehyde	98.985	11.680	144	-2.282	FALSE	[M+K]+
2',3'-Cyclic UMP	329.015	18.712	145	-10.889	FALSE	[M+Na]+
Hexanal	142.123	10.435	146	-4.081	FALSE	[M+C2H3N+H]+
2',3'-cyclic UMP	307.033	20.392	147	-10.877	FALSE	[M+H]+
Pseudouridine-5'-phosphate	307.033	20.392	147	-11.011	FALSE	[M-H2O+H]+
3'-uridylic acid	307.033	20.392	147	-11.011	FALSE	[M-H2O+H]+
Uridine 5'-monophosphate	307.033	20.392	147	-11.011	FALSE	[M-H2O+H]+
D-pantothenic acid	261.145	6.968	150	4.548	FALSE	[M+C2H3N+H]+
Carbamoylphosphate	163.973	3.317	155	-1.685	FALSE	[M+Na]+
Lauric acid	183.174	22.283	159	-13.373	FALSE	[M-H2O+H]+
Biotin	178.123	13.593	160	11.862	FALSE	[M+C2H3N+H]+
Pseudouridine 5'-phosphate	325.043	11.948	161	0.894	TRUE	[M+H]+
3'-uridylic acid	325.043	11.948	161	0.894	TRUE	[M+H]+
Uridine-5'-monophosphate	325.043	11.948	161	0.894	TRUE	[M+H]+
Cifostodine	328.031	3.017	166	-11.231	FALSE	[M+Na]+
Farnesyl diphosphate	424.165	9.216	167	-14.213	FALSE	[M+C2H3N+H]+
UDP-N-acetylmuramoyl-L-alanine	445.229	14.434	171	-1.336	TRUE	[M-H2O+H]+
3-carboxy-cis,cis-muconic acid	169.014	9.050	182	-7.918	FALSE	[M-H2O+H]+
4-carboxymuconolactone	169.014	9.050	182	-7.918	FALSE	[M-H2O+H]+
Glucosamine	180.088	9.405	188	-7.177	FALSE	[M+H]+
(2E)-2-hydroxypenta-2,4-dienoic acid	115.039	6.203	191	-5.572	FALSE	[M+H]+

2-acetolactate	115.039	6.203	191	-5.705	FALSE	[M-H2O+H]+
(S)-4,5-dihydroxypentane-2,3-dione	115.039	6.203	191	-5.705	FALSE	[M-H2O+H]+
4-hydroxy-2-oxopentanoic acid	115.039	6.203	191	-5.705	FALSE	[M-H2O+H]+
3-hydroxy-3-methyl-2-oxobutanoic acid	115.039	6.203	191	-5.705	FALSE	[M-H2O+H]+
2',3'-cyclic UMP	348.060	15.945	204	-8.314	FALSE	[M+C2H3N+H]+
Melibiose	325.113	6.532	207	-13.692	FALSE	[M-H2O+H]+
Sucrose	325.113	6.532	207	-13.692	FALSE	[M-H2O+H]+
Alpha-lactose	325.113	6.532	207	-13.692	FALSE	[M-H2O+H]+
Trehalose	325.113	6.532	207	-13.692	FALSE	[M-H2O+H]+
Salicyl alcohol	125.060	9.976	220	-7.717	FALSE	[M+H]+
4-hydroxybenzyl alcohol	125.060	9.976	220	-7.717	FALSE	[M+H]+
DL-thioctic acid	248.077	6.262	223	-7.903	FALSE	[M+C2H3N+H]+
N-succinyl-L-glutamate	248.077	6.262	223	-10.493	FALSE	[M+H]+
Keto-3-deoxy-D-manno-octulosonic acid	221.066	4.200	231	-9.166	FALSE	[M-H2O+H]+
Biotin sulfoxide	189.112	11.171	233	-2.810	FALSE	[M+2H2O+H]+
Propionaldehyde	117.091	13.104	237	-6.683	FALSE	[2M+H]+
N1-Acetylspermidine	251.185	9.452	238	-8.543	FALSE	[M+C2H3N+Na]+
N8-Acetylspermidine	251.185	9.452	238	-8.543	FALSE	[M+C2H3N+Na]+
Glucosamine	180.088	7.812	239	-7.177	FALSE	[M+H]+
Imidazol-4-one-5-propionic acid	195.017	2.087	258	-7.424	FALSE	[M+K]+
Raffinose	505.177	2.088	258	-20.405	FALSE	[M+H]+
Maltotriose	505.177	2.088	258	-20.405	FALSE	[M+H]+
6-O-(alpha-D-galactopyranosyl)-6-O-(alpha-D-galactopyranosyl)-D-glucopyranose	505.177	2.088	258	-20.405	FALSE	[M+H]+

Table S2. Features matched to PyFBA predicted metabolites in the HILIC run with data acquisition in negative mode.

**Compound**	**Mass to Charge**	**Retention Time (min)**	**Peak Group**	**Isotope Difference (%)**	**Isotope Match**	**Adduct**
Triphosphoric acid	134.894	12.281	2	-0.119	TRUE	[M+Na-2H]-
Palmitic acid	255.232	13.720	3	-17.736	FALSE	[M-H]-
Stearic acid	283.263	13.706	3	-19.942	FALSE	[M-H]-
2-octaprenyl-6-methoxyphenol	705.468	12.053	4	-10.716	FALSE	[M+K-2H]-
L-phenylalanine	164.071	11.412	5	5.311	FALSE	[M-H]-
Tetradecanoic acid	227.200	11.216	8	0.692	TRUE	[M-H]-
Sulfurous acid	80.965	11.080	9	-0.949	TRUE	[M-H]-
Methyl alpha-D-glucopyranoside	253.093	9.265	11	15.183	FALSE	[M+CH3COO(-)]-
Acetic acid	59.014	1.488	12	-2.294	FALSE	[M-H]-
Glycolaldehyde	59.014	1.488	12	-2.294	FALSE	[M-H]-
3-methyl-2-oxobutanoic acid	115.040	1.506	12	4.338	FALSE	[M-H]-
2-hydroxy-3-(phosphonooxy)propyl laurate	413.196	1.500	12	13.970	FALSE	[M+CH3COO(-)]-
Diadenosine tetraphosphate	553.035	17.033	16	-1.303	TRUE	[M+CH3COO(-)]-
1-Stearoyl-sn-glycero-3-phosphate	475.189	11.993	17	-2.516	FALSE	[M+K-2H]-
Tetradecanoic acid	227.200	13.156	18	-15.530	FALSE	[M-H]-
3-oxoadipyl-CoA	656.204	16.238	22	30.619	FALSE	[M+Cl(-)]-
D-Methionine	208.064	14.048	23	4.540	FALSE	[M+CH3COO(-)]-
L-methionine	208.064	14.048	23	4.540	FALSE	[M+CH3COO(-)]-
3-oxopropanoic acid	87.008	14.029	23	-3.412	FALSE	[M-H]-
Pyruvic acid	87.008	14.029	23	-3.412	FALSE	[M-H]-
(2S)-2-Isopropylmalate	87.008	14.029	23	-7.911	FALSE	[M-2H]-
3-isopropylmalate	87.008	14.029	23	-7.911	FALSE	[M-2H]-
Lauric acid	199.169	13.510	27	-13.324	FALSE	[M-H]-
2-octaprenyl-3-methyl-5-hydroxy-6-methoxy-1,4-benzoquinone	747.515	12.084	30	-11.404	FALSE	[M+Cl(-)]-

Benzoic acid	121.029	12.112	30	-7.717	FALSE	[M-H]-
L-fuculose	163.060	1.535	31	1.171	FALSE	[M-H]-
L-rhamnose	163.060	1.535	31	1.171	FALSE	[M-H]-
L-fucose	163.060	1.535	31	1.171	FALSE	[M-H]-
6-deoxy-L-fructose	163.060	1.535	31	1.171	FALSE	[M-H]-
3-hydroxyisobutyric acid	163.060	1.535	31	5.735	FALSE	[M+CH3COO(-)]-
(R)-3-hydroxybutanoic acid	163.060	1.535	31	5.735	FALSE	[M+CH3COO(-)]-
dUTP	526.986	14.317	34	5.935	FALSE	[M+CH3COO(-)]-
(S)-3-hydroxyoctanoyl-CoA	900.777	14.396	34	13.669	FALSE	[M+K-2H]-
Glycerol	112.985	9.835	37	-3.469	FALSE	[M+Na-2H]-
Guanine	150.041	9.820	37	0.080	TRUE	[M-H]-
2-octaprenyl-3-methyl-5-hydroxy-6-methoxy-1,4-benzoquinone	747.514	10.146	38	-12.160	FALSE	[M+Cl(-)]-
Hypoxanthine	135.031	10.149	38	-0.079	TRUE	[M-H]-
Glycolic acid	135.031	10.149	38	6.843	FALSE	[M+CH3COO(-)]-
Coenzyme Q8	761.531	10.141	38	-11.802	FALSE	[M+Cl(-)]-
Ethanol	82.954	10.233	38	-2.290	FALSE	[M+K-2H]-
2-octaprenyl-6-methoxyphenol	705.469	10.275	38	-51.687	FALSE	[M+K-2H]-
3-oxotetradecanoyl-CoA	467.409	10.153	38	-41.960	FALSE	[M-2H]-
Phosphoric acid	96.969	31.203	39	-0.198	TRUE	[M-H]-
Carbonic acid	96.969	31.203	39	-1.217	TRUE	[M+Cl(-)]-
L-dihydroorotic acid	194.946	31.231	39	-5.874	FALSE	[M+K-2H]-
Diphosphoric acid	176.935	31.118	39	-0.324	TRUE	[M-H]-
2-keto-3-deoxy-6-phosphogluconate	294.926	31.065	44	-6.972	FALSE	[M+K-2H]-
6-phospho-2-dehydro-3-deoxy-D-galactonic acid	294.926	31.065	44	-6.972	FALSE	[M+K-2H]-
6-phosphogluconolactone	294.926	31.065	44	-6.972	FALSE	[M+K-2H]-
Phosphoric acid	156.990	20.141	46	6.664	FALSE	[M+CH3COO(-)]-
Uracil	111.020	2.661	47	-0.191	TRUE	[M-H]-

Lauric acid	199.169	11.358	48	0.091	TRUE	[M-H]-
Glyoxylic acid	72.993	11.135	51	-2.309	FALSE	[M-H]-
L-arabino-1,4-lactone	72.993	11.135	51	-5.705	FALSE	[M-2H]-
Palmitic acid	277.179	11.049	54	-17.747	FALSE	[M+Na-2H]-
2-octaprenyl-6-methoxyphenol	689.493	6.637	55	-28.748	FALSE	[M+Na-2H]-
Stearic acid	283.263	4.434	57	-19.942	FALSE	[M-H]-
Cifostodine	304.033	21.551	61	-0.323	TRUE	[M-H]-
Cytosine	110.035	21.546	61	-5.534	FALSE	[M-H]-
D-glutamic acid	146.045	22.096	62	-0.402	TRUE	[M-H]-
L-glutamic acid	146.045	22.096	62	-0.402	TRUE	[M-H]-
O-acetyl-L-serine	146.045	22.096	62	-0.402	TRUE	[M-H]-
4-aminobutyric acid	102.055	22.093	62	-0.085	TRUE	[M-H]-
L-pyroglutamic acid	128.035	22.093	62	0.401	TRUE	[M-H]-
3-hydroxy-L-1-pyrroline-5-carboxylate	128.035	22.093	62	0.401	TRUE	[M-H]-
2,3-dihydroxyisovaleric acid	184.001	22.088	62	-3.293	FALSE	[M+CH3COO(-)]-
2-deoxy-d-ribofuranose	184.001	22.088	62	-3.293	FALSE	[M+CH3COO(-)]-
O-phospho-L-serine	184.001	22.088	62	-3.941	FALSE	[M-H]-
Sulfurous acid	80.965	13.980	64	-0.949	TRUE	[M-H]-
dUTP	526.985	16.787	67	6.347	FALSE	[M+CH3COO(-)]-
Palmitic acid	255.231	5.297	69	-17.736	FALSE	[M-H]-
Nitric acid	61.988	1.428	72	-0.506	TRUE	[M-H]-
D-glyceric acid	165.040	1.420	72	-1.191	TRUE	[M+CH3COO(-)]-
L-tryptophan	239.059	1.390	72	5.861	FALSE	[M+Cl(-)]-
5'-methylthio-D-ribose	239.059	1.390	72	13.392	FALSE	[M+CH3COO(-)]-
Glycerol	112.985	1.401	72	-3.469	FALSE	[M+Na-2H]-
Glycyl-L-glutamine	224.028	1.403	72	-8.992	FALSE	[M+Na-2H]-
2-octaprenyl-6-methoxyphenol	689.494	3.598	75	-27.333	FALSE	[M+Na-2H]-
(3S)-3-hydroxyadipyl-CoA	660.162	16.057	76	40.892	FALSE	[M+K-2H]-
Sulfuric acid	96.960	28.652	80	-0.987	TRUE	[M-H]-

2-ketoglutaric acid	145.013	28.687	80	-5.671	FALSE	[M-H]-
D-erythrose 4-phosphate	259.022	28.669	80	-2.419	FALSE	[M+CH3COO(-)]-
Beta-D-glucose-1-phosphate	259.022	28.669	80	-6.983	FALSE	[M-H]-
D-Fructose 6-phosphate	259.022	28.669	80	-6.983	FALSE	[M-H]-
D-fructofuranose 1-phosphate	259.022	28.669	80	-6.983	FALSE	[M-H]-
Alpha-D-mannose 1-phosphate	259.022	28.669	80	-6.983	FALSE	[M-H]-
Inositol 3-phosphate	259.022	28.669	80	-6.983	FALSE	[M-H]-
1D-myo-inositol 4-phosphate	259.022	28.669	80	-6.983	FALSE	[M-H]-
Inositol 1-phosphate	259.022	28.669	80	-6.983	FALSE	[M-H]-
D-arabino-3-hexulose 6-phosphate	259.022	28.669	80	-6.983	FALSE	[M-H]-
Galactose-1-phosphate	259.022	28.669	80	-6.983	FALSE	[M-H]-
Mannose 6-phosphate	259.022	28.669	80	-6.983	FALSE	[M-H]-
D-glucopyranose 6-phosphate	259.022	28.669	80	-6.983	FALSE	[M-H]-
Glucose-1-phosphate	259.022	28.669	80	-6.983	FALSE	[M-H]-
Beta-D-Glucose 6-phosphate	259.022	28.669	80	-6.983	FALSE	[M-H]-
D-tagatose 6-phosphate	259.022	28.669	80	-6.983	FALSE	[M-H]-
D-galactopyranose 1-phosphate	259.022	28.669	80	-6.983	FALSE	[M-H]-
sn-Glycerol 3-phosphate	171.006	28.652	80	-3.583	FALSE	[M-H]-
2-octaprenyl-6-methoxyphenol	689.493	9.128	83	-25.860	FALSE	[M+Na-2H]-
Folic acid	500.154	15.834	91	41.735	FALSE	[M+CH3COO(-)]-
Palmitic acid	255.232	2.140	92	-17.736	FALSE	[M-H]-
Guanine	150.041	9.009	94	0.080	TRUE	[M-H]-
Acetic acid	59.013	24.451	98	0.160	TRUE	[M-H]-
Glycolaldehyde	59.013	24.451	98	0.160	TRUE	[M-H]-
2-octaprenyl-6-methoxyphenol	689.493	1.605	102	-26.480	FALSE	[M+Na-2H]-
L-allysine	204.087	13.665	103	-4.819	FALSE	[M+CH3COO(-)]-
6-amino-2-oxohexanoic acid	204.087	13.665	103	-4.819	FALSE	[M+CH3COO(-)]-
Propionic acid	73.029	27.660	109	0.347	TRUE	[M-H]-
(S)-lactaldehyde	73.029	27.660	109	0.347	TRUE	[M-H]-

Lactaldehyde	73.029	27.660	109	0.347	TRUE	[M-H]-
Succinic acid	117.019	27.651	109	0.819	TRUE	[M-H]-
Guanine	150.041	22.696	112	-7.352	FALSE	[M-H]-
Nicotinamide dinucleotide	662.100	22.717	112	0.289	TRUE	[M-H]-
Glycerol	112.985	5.613	113	0.001	TRUE	[M+Na-2H]-
Tartronate semialdehyde	103.003	28.046	118	-3.450	FALSE	[M-H]-
3-hydroxypyruvic acid	103.003	28.046	118	-3.450	FALSE	[M-H]-
Malonic acid	103.003	28.046	118	-3.450	FALSE	[M-H]-
NADH	664.118	12.563	127	-26.140	FALSE	[M-H]-
Altronic acid	195.050	20.764	128	-0.396	TRUE	[M-H]-
D-galactonic acid	195.050	20.764	128	-0.396	TRUE	[M-H]-
Gluconic acid	195.050	20.764	128	-0.396	TRUE	[M-H]-
D-mannonic acid	195.050	20.764	128	-0.396	TRUE	[M-H]-
L-arginine	195.050	20.764	128	-1.716	FALSE	[M+Na-2H]-
dUTP	526.985	15.198	140	5.970	FALSE	[M+CH3COO(-)]-
dUTP	502.942	14.620	144	0.604	TRUE	[M+Cl(-)]-
UDP-glucose	565.046	28.326	162	0.370	TRUE	[M-H]-
Uridine diphosphogalactose	565.046	28.326	162	0.370	TRUE	[M-H]-
Pantothenylcysteine 4'-phosphate	401.076	28.312	162	-15.117	FALSE	[M-H]-
2-octaprenyl-6-methoxyphenol	689.493	2.573	163	-26.439	FALSE	[M+Na-2H]-
2-Deoxyadenosine-5-monophosphate	282.083	11.295	167	28.403	FALSE	[M+CH3COO(-)]-
N-acetyl-L-glutamyl 5-phosphate	328.044	18.916	170	7.199	FALSE	[M+CH3COO(-)]-
Adenine	134.047	18.922	170	-0.150	TRUE	[M-H]-
Glycine	134.047	18.922	170	6.772	FALSE	[M+CH3COO(-)]-
Fumaric acid	115.003	27.777	171	1.408	FALSE	[M-H]-
L-malic acid	133.014	27.787	171	-0.476	TRUE	[M-H]-
D-(+)-malic acid	133.014	27.787	171	-0.476	TRUE	[M-H]-
Glyoxylic acid	133.014	27.787	171	4.088	FALSE	[M+CH3COO(-)]-

Methylglyoxal	71.013	27.771	171	-3.374	FALSE	[M-H]-
2-octaprenyl-6-methoxyphenol	727.564	1.369	173	-49.394	FALSE	[M+CH3COO(-)]-
Ubiquinol-8	727.564	1.369	173	-53.958	FALSE	[M-H]-
Uridine	243.061	2.888	175	0.389	TRUE	[M-H]-
2-octaprenyl-6-methoxyphenol	689.494	4.290	179	-25.864	FALSE	[M+Na-2H]-
2-octaprenyl-6-methoxyphenol	689.493	7.310	186	-27.500	FALSE	[M+Na-2H]-
Uracil	111.020	1.995	189	0.277	TRUE	[M-H]-
Uracil	111.020	19.628	191	1.367	FALSE	[M-H]-
Isopentenyl pyrophosphate	305.018	19.637	191	-3.534	FALSE	[M+CH3COO(-)]-
DMAPP	305.018	19.637	191	-3.534	FALSE	[M+CH3COO(-)]-
2',3'-Cyclic UMP	305.018	19.637	191	-10.900	FALSE	[M-H]-
Sulfurous acid	80.965	7.392	208	-0.949	TRUE	[M-H]-
Betaine	116.071	11.521	209	5.575	FALSE	[M-H]-
L-valine	116.071	11.521	209	5.575	FALSE	[M-H]-
Palmitic acid	255.232	16.349	213	-0.699	TRUE	[M-H]-
Stearic acid	283.263	16.349	213	-0.072	TRUE	[M-H]-
Benzoic acid	121.029	3.174	223	10.556	FALSE	[M-H]-
Pseudouridine 5'-phosphate	323.028	29.525	224	-10.961	FALSE	[M-H]-
3'-uridylic acid	323.028	29.525	224	-10.961	FALSE	[M-H]-
Uridine 5'-monophosphate	323.028	29.525	224	-10.961	FALSE	[M-H]-
Glyoxylic acid	72.993	14.150	227	-2.309	FALSE	[M-H]-
L-arabino-1,4-lactone	72.993	14.150	227	-5.705	FALSE	[M-2H]-
5'-Cytidylic acid	322.044	29.692	233	2.109	FALSE	[M-H]-
Cytidine 3'-monophosphate	322.044	29.692	233	2.109	FALSE	[M-H]-
Methanesulfonic acid	94.980	23.302	234	-2.052	FALSE	[M-H]-
Glyceraldehyde-3-phosphate	229.011	28.770	245	-1.278	TRUE	[M+CH3COO(-)]-
Dihydroxyacetone phosphate	229.011	28.770	245	-1.278	TRUE	[M+CH3COO(-)]-
D-xylulose 5-phosphate	229.011	28.770	245	-5.842	FALSE	[M-H]-
Ribose 1-phosphate	229.011	28.770	245	-5.842	FALSE	[M-H]-
L-xylulose 5-phosphate	229.011	28.770	245	-5.842	FALSE	[M-H]-

D-ribose 5-phosphate	229.011	28.770	245	-5.842	FALSE	[M-H]-
D-arabinose 5-phosphate	229.011	28.770	245	-5.842	FALSE	[M-H]-
[(3R,4S)-2,3,4-Trihydroxyoxolan-2-yl]methyl dihydrogen phosphate	229.011	28.770	245	-5.842	FALSE	[M-H]-
Ribulose 5-phosphate	229.011	28.770	245	-5.842	FALSE	[M-H]-
L-ribulose 5-phosphate	229.011	28.770	245	-5.842	FALSE	[M-H]-
Tricarballylic acid	211.001	28.782	245	-6.800	FALSE	[M+Cl(-)]-
Methanesulfonic acid	94.980	24.967	246	-2.052	FALSE	[M-H]-
2-octaprenyl-6-methoxyphenol	689.494	5.173	253	-28.447	FALSE	[M+Na-2H]-
Uridine 5'-diphosphate	402.994	36.262	254	-11.087	FALSE	[M-H]-
Beta-D-glucose 1-phosphate	259.021	29.346	256	-6.983	FALSE	[M-H]-
D-fructose 6-phosphate	259.021	29.346	256	-6.983	FALSE	[M-H]-
D-fructofuranose 1-phosphate	259.021	29.346	256	-6.983	FALSE	[M-H]-
Alpha-D-mannose 1-phosphate	259.021	29.346	256	-6.983	FALSE	[M-H]-
Inositol 3-phosphate	259.021	29.346	256	-6.983	FALSE	[M-H]-
1D-myo-inositol 4-phosphate	259.021	29.346	256	-6.983	FALSE	[M-H]-
inositol 1-phosphate	259.021	29.346	256	-6.983	FALSE	[M-H]-
D-arabino-3-hexulose 6-phosphate	259.021	29.346	256	-6.983	FALSE	[M-H]-
Galactose-1-phosphate	259.021	29.346	256	-6.983	FALSE	[M-H]-
Mannose 6-phosphate	259.021	29.346	256	-6.983	FALSE	[M-H]-
D-glucopyranose 6-phosphate	259.021	29.346	256	-6.983	FALSE	[M-H]-
Glucose-1-phosphate	259.021	29.346	256	-6.983	FALSE	[M-H]-
Beta-D-glucose 6-phosphate	259.021	29.346	256	-6.983	FALSE	[M-H]-
D-tagatose 6-phosphate	259.021	29.346	256	-6.983	FALSE	[M-H]-
D-galactopyranose 1-phosphate	259.021	29.346	256	-6.983	FALSE	[M-H]-
dADP	362.049	32.533	267	-1.169	TRUE	[M+CH3COO(-)]-
Cytidine 5'-diphosphate	402.009	36.113	269	-11.429	FALSE	[M-H]-
dTDP-4-keto-L-rhamnose	567.004	19.073	277	-18.887	FALSE	[M+Na-2H]-

dTDP-4-dehydro-6-deoxy-alpha-D-glucose	567.004	19.073	277	-18.887	FALSE	[M+Na-2H]-
L-arabino-1,4-lactone	147.029	27.475	279	-5.694	FALSE	[M-H]-
dTDP-L-rhamnose	547.072	27.108	285	-18.899	FALSE	[M-H]-
UDP-N-acetylmannosamine	606.072	27.882	287	-20.435	FALSE	[M-H]-
UDP-N-acetylglucosamine	606.072	27.882	287	-20.435	FALSE	[M-H]-
UDP-N-acetylgalactosamine	606.072	27.882	287	-20.435	FALSE	[M-H]-
Folic acid	500.154	12.620	291	43.062	FALSE	[M+CH3COO(-)]-
N-Succinyl-L-glutamate	246.061	33.672	303	-10.516	FALSE	[M-H]-

4. Materials and Methods

4.1 MS_FBA parameters

The MS_FBA function evaluates XCMS Online results against a custom compound list using the following parameters: **xcms_tsvfile**, **files**, **file_prefixes**, **compounds_tsv**, **ion_mode**, **pvalue**, **ppm** and **isotol**. These are the nine variables that must be set for the MS_FBA function to complete:

1. **xcms_tsvfile**: This is located in the results folder downloaded after the XCMS Online run is completed; the exact title will be XCMS.annotated.diffreport.tsv.
2. **files**: This needs to be a character vector of the file names that were produced from the mass spectrometer. These are the same files that are analyzed using XCMS Online. It is easiest to have the files in the working directory, so the path is not required every time (e.g. M1, M2, M3...etc).
3. **file_prefixes**: This is also a character vector, but it is just the prefixes for how you split up your sample classes (e.g. M, L, S).
4. **compounds_tsv**: This is the tsv of the expected compounds that you are wishing to compare the XCMS Online results against. This tab-separated-values (tsv) file must be formatted with the fields: "compound", "monoisotopic mass", and "molecular formula" followed by the compounds, each separated by tabs, and with each record separated by a newline. This file can be created from any flux balance analysis program, manually from any list of compounds, or directly in the PyFBA [3] script that is provided.
5. **ion_mode**: This is either 'N' or 'P' for whether the experiment was run in negative or positive mode. This variable alters the potential adducts list (see **Supplemental Table S3**).
6. **pvalue**: This variable determines the features that should be considered as a viable feature from the XCMS Online results, default is 0.05. If the *P*-value is less than the input threshold then the feature will be included in the search, otherwise it will be disregarded.
7. **ppm**: This is the mass deviation in parts per million is used to create a mass matching window for features to peaks in the raw files and features to potential compounds, default is set to 5 ppm.
8. **rtmulti**: The retention time multiplier is responsible for the retention time window created. Since XCMS Online performs peak alignment, it is important to expand the retention time window to ensure that the top of the peak is always found for each feature in the raw data files. The larger the window the longer the program takes to run but the more certain the peak maximum will be correctly identified. The retention time window

is created by taking the RT difference from XCMS Online results and multiplying it by the rtmulti value, then adding the resulting time window on either side of the pre-existing window (e.g. if the RT window was from 1.9 to 2 minutes and the rtmulti was 2 the new RT window would be 1.7 to 2.2 minutes).

9. **isotol**: The isotope ratio deviation tolerance in percent. This is the cut-off for whether the metabolite feature as a TRUE or FALSE isotope match within the ranked list.

Table S3. Adducts with the ionization mode that they are present in and the mass change that occurs to the compounds mass (M).

**Adduct**	**Ionization mode**	**Mass change**
$[M+H]^{+}$	Positive	M + 1.007825
$[M+Na]^{+}$	Positive	M + 22.98977
$[M+K]^{+}$	Positive	M + 38.96371
$[2M+H]^{+}$	Positive	(2 x M) + 1.007825
$[M-H2O+H]^{+}$	Positive	M - 17.00274
$[M+2H2O+H]^{+}$	Positive	M + 37.02896
$[M+C2H3N+H]^{+}$	Positive	M + 42.03437
$[M+C2H3N+Na]^{+}$	Positive	M + 64.01632
$[M+2Na-H]^{+}$	Positive	M + 44.97172
$[M+2H]^{2+}$	Positive	(M + 2.05165) / 2
$[M+H+Na]^{2+}$	Positive	(M + 23.9976) / 2
$[M-H]^{-}$	Negative	M - 1.007825
$[M+Na-2H]^{-}$	Negative	M +20.93812
$[M+K-2H]^{-}$	Negative	M +36.91206
$[M+CH3COO]^{-}$	Negative	M + 59.013853
$[M+Cl]^{-}$	Negative	M + 34.968304
$[M-2H]^{2-}$	Negative	(M - 2.05165) / 2

4.1 XCMS Online method parameters for mass spectral analysis

Modified versions of the generic "UPLC/Bruker Q-TOF pos" parameter set were used to perform multigroup analyses of the three sets of samples at different time points for each LCMS condition. Listed below in **Supplemental Table S4** are the specific method parameters that have been changed compared to the default settings. It is not imperative to have adducts set in this annotation tab as MS_FBA independently looks for adducts listed in **Supplemental Table S3**.

Table S4. XCMS Online [1] method parameters "UPLC/Bruker Q-TOF pos" method for each LCMS condition.

**Parameters**		**Reverse Phase, Positive Mode**	**HILIC, Negative Mode**
General tab	Polarity	positive	negative
Feature detection tab	Minimum peak width	5	10
	Maximum peak width	20	50
Alignment tab	bw	5	10
Statistics tab	p-value threshold (highly significant features)	0.05	0.05
	p-value threshold (significant features)	0.1	0.1
Annotation tab	ppm	5	10
Identification tab*	adducts	[M+H]+<br>[M+Na]+<br>[M+H-H2O]+<br>[M+H-2H2O]+<br>[M+K]+<br>[M+ACN+H]+<br>[M+ACN+Na]+<br>[M+2Na-H]+<br>[M+2H]2+<br>[M+H+Na]2+<br>[M+2H+Na]3+<br>[M+2Na]2+<br>[M+2Na+H]3+<br>[M+]+	[M-H]-
	Sample biosource	CFRE742730-HMP	CFRE742730-HMP

*Identification of adducts at this stage performs matching with the METLIN database[2]

References

1. Gowda, H.; Ivanisevic, J.; Johnson, C.H.; Kurczy, M.E.; Benton, H.P.; Rinehart, D.; Nguyen, T.; Ray, J.; Kuehl, J.; Arevalo, B.; et al. Interactive XCMS Online: Simplifying Advanced Metabolomic Data Processing and Subsequent Statistical Analyses. *Anal. Chem.* **2014**, *86*, 6931–6939.
2. Zhu, Z.J.; Schultz, A.W.; Wang, J.; Johnson, C.H.; Yannone, S.M.; Patti, G.J.; Siuzdak, G. Liquid chromatography quadrupole time-of-flight mass spectrometry characterization of metabolites guided by the METLIN database. *Nat. Protoc.* **2013**, *8*, 451–460.
3. Cuevas, D.A.; Edirisinghe, J.; Henry, C.S.; Overbeek, R.; O'Connell, T.G.; Edwards, R.A. From DNA to FBA: How to Build Your Own Genome-Scale Metabolic Model. *Front. Microbiol.* **2016**, *7*, 907.

© 2020 by the authors. Submitted for possible open access publication under the terms and conditions of the Creative Commons Attribution (CC BY) license (http://creativecommons.org/licenses/by/4.0/).
